# *ABCD4* is associated with mammary gland development in mammals

**DOI:** 10.1186/s12864-024-10398-9

**Published:** 2024-05-20

**Authors:** Xiaoli Guo, Chengcheng Zhao, Ruifei Yang, Yuzhe Wang, Xiaoxiang Hu

**Affiliations:** 1 State Key Laboratory of Swine and Poultry Breeding Industry & Guangdong Provincial Key Laboratory of Animal Breeding and Nutrition &, Institute of Animal Science, Guangdong Academy of Agricultural Sciences, Guangzhou, 510640 China; 2 State Key Laboratory of Animal Biotech Breeding, College of Biological Sciences, China Agricultural University, Beijing, 100193 China

**Keywords:** *ABCD4*, HC11, Mammary gland development, *PRLR*

## Abstract

**Background:**

Mammary gland development is a critical process in mammals, crucial for their reproductive success and offspring nourishment. However, the functional roles of key candidate genes associated with teat number, including *ABCD4*, *VRTN*, *PROX2*, and *DLST*, in this developmental process remain elusive. To address this gap in knowledge, we conducted an in-depth investigation into the dynamic expression patterns, functional implications, and regulatory networks of these candidate genes during mouse mammary gland development.

**Results:**

In this study, the spatial and temporal patterns of key genes were characterized in mammary gland development. Using time-series single-cell data, we uncovered differences in the expression of *A bcd4*, *Vrtn*, *Prox2*, and *Dlst* in cell population of the mammary gland during embryonic and adult stages, while *Vrtn* was not detected in any cells. We found that only overexpression and knockdown of *Abcd4* could inhibit proliferation and promote apoptosis of HC11 mammary epithelial cells, whereas *Prox2* and *Dlst* had no significant effect on these cells. Using RNA-seq and qPCR, further analysis revealed that *Abcd4* can induce widespread changes in the expression levels of genes involved in mammary gland development, such as *Igfbp3*, *Ccl5*, *Tlr2*, and *Prlr*, which were primarily associated with the MAPK, JAK-STAT, and PI3K-AKT pathways by functional enrichment.

**Conclusions:**

These findings revealed *ABCD4* as a candidate gene pivotal for regulating mammary gland development and lactation during pregnancy by influencing *PRLR* expression.

**Supplementary Information:**

The online version contains supplementary material available at 10.1186/s12864-024-10398-9.

## Introduction

Mammalian mammary gland development is essential for offspring survival and reproductive success, enabling the production and provision of milk, which serves as a critical source of nutrition and immune protection for newborns. Epithelial cell proliferation and apoptosis are fundamental processes that orchestrate the intricate development of the mammary gland across various reproductive stages [1, 2]. Beginning in embryonic development, epithelial cell proliferation drives the formation of mammary placodes and subsequent bud invagination [3]. Hormonal cues during puberty stimulate epithelial cell proliferation, resulting in the elongation and branching of ductal networks [4]. Elevated epithelial cell proliferation during pregnancy forms alveolar structures necessary for milk synthesis, while lactation involves ongoing proliferation to meet heightened milk demand [5, 6]. Subsequently, involution entails significant epithelial cell apoptosis, facilitating glandular regression and returning to a quiescent state [2, 7]. This delicate balance between epithelial cell proliferation and apoptosis is vital for establishing functional mammary tissue, ensuring successful milk production to support offspring growth and survival. Therefore, understanding of the developmental processes of the mammary gland and the underlying molecular mechanisms holds significant importance for enhancing animal production efficiency and genetic advancements.

Using a low-coverage whole-genome sequencing (LCS) approach, we identified a robust quantitative trait locus (QTL) associated with total teat number on chromosome 7 [8], which explains a significant proportion of phenotypic variation and corroborates findings from previous studies [9–11]. Subsequent fine-mapping efforts delineated this region into two narrow linkage disequilibrium (LD) blocks housing four candidate genes: *ABCD4*, *VRTN*, *PROX2*, and *DLST* [8]. However, there is currently insufficient evidence to suggest their involvement in mammary gland development, necessitating thorough investigation into their functions. Notably, studies indicate parallels between piglet and mouse mammary gland morphological changes across embryonic, pubertal, and gestational stages [12, 13]. Given the conservation of *ABCD4*, *VRTN*, *PROX2*, and *DLST* in mammals (Supplementary Figure S1-S4), and the extensive understanding of mouse mammary gland morphogenesis [14, 15], exploring the roles of these genes in mouse mammary development could help confirm and narrow down the range of functional genes that influence pig teat number.

To systematically identify these candidate genes potentially involved in mammary gland development, we characterized the expression profiles of four genes across mammary gland cell types in mice using the single-cell RNA sequencing (scRNA-seq) dataset. Furthermore, we revealed the gene function and regulatory network of *Abcd4* in mammary epithelial cells of mice through cell proliferation and apoptosis, as well as RNA-seq analysis. Through our study, we aimed to elucidate their contributions to mammary gland morphogenesis and lactation, shedding light on essential mechanisms underlying mammary development in mammals.

## Material and methods

### Analysis of scRNA-seq data

To delineate the expression pattern of candidate genes in mammary gland cells, we utilized a published mouse scRNA-seq dataset [16]. Raw reads were aligned to the mouse genome sequence (GRCm38) with Cell Ranger (v.5.0.1). Data quality control was performed with Seurat (v.3.0) [17]. The raw count of each library for genes expressed in > 3 cells and cells with > 200 detected genes was used for downstream analyses. Matrices were merged, excluding cells with > 2,500 expressed genes and mitochondrial gene percentages > 5%. The expression matrix was normalized and linearly scaled using the NormalizeData and ScaleData functions. Principal component analysis (PCA) was performed with the RunPCA function based on 2,000 genes. Subsequently, cell clustering analysis was carried out using the FindNeighbors and FindClusters functions. For downstream visualization using the uniform manifold approximation and projection (UMAP) technique, the top 20 dimensions were selected. The data was integrated and analyzed following the pipeline outlined in the previous study [16].

### Mice and sample collections

All mice used in this study were C57BL/6 wild-type mice procured from Vital River Laboratories (Beijing, China). The mice were housed in environmentally controlled rooms on a 12-h light–dark cycle and had free access to food and water. Cervical dislocation was employed for euthanasia. Mammary tissues were collected during 11 critical periods of mammary gland development, including late embryonic stages: embryonic E16.5 (*n* = 6) and E18.5 (*n* = 4); postnatal stages: postnatal 1 day (*n* = 5), 1 week (*n* = 3) and 1 month (*n* = 5); mid to late pregnancy stages: 13.5 days (*n* = 3), 16.5 days (*n* = 4) and 18.5 days (*n* = 5) of pregnancy; lactation period stages: 1 day (*n* = 3), 1 week (*n* = 5) and 1 month (*n* = 3) of lactation. Skin tissues containing the mammary gland were meticulously collected and promptly frozen in liquid nitrogen. A small amount of head tissue was used for sex identification with a TransDirect Animal Tissue PCR Kit (TransGen Biotech, Beijing, China, AS201-02). Sex identification relied on the expression of the male-specific gene Sry, and only the mammary glands from female mice were retained for subsequent investigations.

### Cell culture and treatment

HC11 mouse mammary epithelial cells were cultured in RPMI-1640 medium with 10% fetal bovine serum (FBS), 5 mg/mL insulin (Sigma, I5500), 10 ng/mL epidermal growth factor (EGF; GIBCO, Grand Island, NY, USA, PGH0315), and 5 mg/mL gentamycin sulfate. Cells were incubated in a cell culture incubator with 5% carbon dioxide (CO_2_) at 37 °C. Transfection was performed when cells reached 60% confluency using FuGENE HD transfection reagent (Promega, Madison, WI, USA, E2311). Each treatment group was performed on the same plate with at least three replicates.

### Plasmid construction and overexpression

The entire coding region of each candidate gene was amplified using forward primers containing the EcoRI site and a reverse primer containing the NotI site. Phanta Super Fidelity DNA Polymerase (Vazyme, Nanjing, China, P505) was used for high-fidelity amplification. PCR fragments were generated by double enzyme digestion and ligated with pcDNA3.1 expression vectors using T4 DNA ligase (NEB, Beverly, MA, USA, M0201), followed by transformation into DH5α competent cells (Cwbio, Beijing, China, CW0808). Sequencing verified all plasmid constructs to exclude mutations, and these plasmids were then transfected into HC11 cells. After 24–48 h, cells were collected for further analysis or experiments.

### Cell proliferation and apoptosis analysis

Cell proliferation was evaluated using the BeyoClick™ EdU (5-ethynyl-2’-deoxyuridine) Cell Proliferation Kit with Alexa Fluor 488 (Beyotime, Shanghai, China, C0071) according to the manufacturer’s protocol. HC11 cells were seeded into 6-well plates. 48 h post-transfection, cells were incubated in a medium containing 10 μM EdU solution at 37 °C for 2 h. EdU-abeled cells were digested with trypsin and transferred to a 1.5 ml centrifuge tube. Subsequently, cells were fixed with 4% paraformaldehyde for 15 min, washed twice, and incubation with 500 μL permeation solution (Beyotime, Shanghai, China, P0106) for 15 min. After two additional washes, cells were incubated with Click Additive Solution for 30 min in the dark. Following three washes, cells were analyzed by flow cytometry within 1 h.

The apoptosis rate was asswssed using the Annexin V-FITC Detection Kit (Beyotime, Shanghai, China, C1062). Cells were seeded into 12–well tissue culture plates. After treatment, 5 × 10^4^ cells/sample were collected, washed with PBS, and resuspended in 195 μL Annexin V-FITC binding buffer. Then, 5 μL Annexin V-FITC and 10 μL propidium iodide (PI) were added to the buffer, and the cells were incubated at room temperature in the dark for 10–20 min. Subsequently, cells were analyzed by flow cytometry within 1 h, and data were processed using FlowJo software.

### RNA extraction and quantitative real-time PCR

Total RNA from the cultured cells or tissues was isolated using a Total RNA Kit I (Omega, Norcross, GA, R6834), followed by conversion to complementary DNA (cDNA) with a PrimeScript™ RT Reagent Kit (TaKaRa, Tokyo, Japan, RR047A). qRT-PCR was conducted on an ABI 7500 using TB Green® Premix Ex Taq™ (TaKaRa, Tokyo, Japan, RR420A) in accordance with the manufacturer’s instructions. The *Gapdh* gene served as the internal control, and the primer sequences for all candidate genes are listed in Supplementary Table S1.

### RNA-seq analysis

Six replicates were performed for RNA-seq analysis. The RNA library was constructed following Illumina library preparation protocols and was sequenced on the DNB-T7 platform. Raw reads were filtered with Trimmomatic (v.0.39) [18] and then mapped to the mouse reference genome (GRCm38) using HISAT2 (v2.0.5) [19]. Htseq-count (v.0.12.3) generated the count matrix. Differentially expressed genes (DEGs) were identified with DESeq2 (v1.32.0) [20] using thresholds of baseMean (mean of normalized counts of all samples) > 10, *padj* value (adjusted *P* value) < 0.05 and |log_2_ (Fold change)|> 1. Gene ontology (GO) and Kyoto Encyclopedia of Genes and Genomes (KEGG) analysis were performed with the Metascape online tool (http://metascape.org).

### Statistical analysis

Statistical analyses were carried out using SPSS 22.0 (SPSS, Chicago, IL, USA). The data are expressed as mean ± SD (standard deviation) and were analyzed using a two-tailed Student’s t-test. At least three replicates were conducted in multiple independent experiments, and differences were considered statistically significant at a *P*-value < 0.05.

## Results

### Expression of candidate genes in the mammary gland

**Fig. 1:**
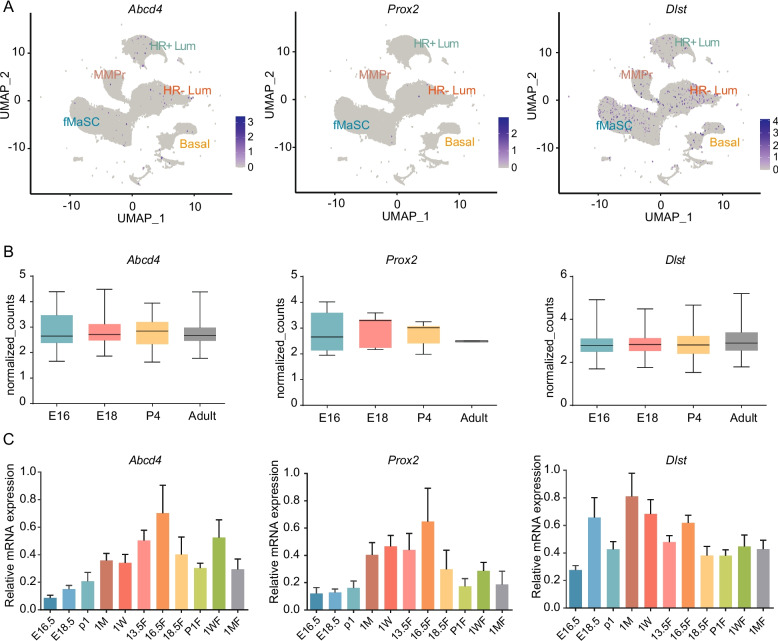
Expression of candidate genes in the mammary gland. **A** Single-cell sequencing analysis of the relative expression of candidate genes in subpopulations. The blue dots indicate the cells with gene expression. **B** The expression of candidate genes in E16, E18, P4 and adult in mouse mammary gland cells. **C** qPCR analysis of candidate genes mRNA expression at different stages of mammary gland development. Different colors in the figure represent different stages. The values are the means ± SEMs for at least three independent experiments

To characteristic the expression profiles of the four candidate genes in mammary cells, we analyzed scRNA-seq data from mice mammary gland published by Giraddi et al. [16]. The scRNA-seq data include four developmental periods: embryonic day 16 (E16), E18, postnatal day 4 (P4), and the adult stage (Adu). All cells were clustered into 22 cell clusters using UMAP (Supplementary Figure S5). According to the expression of classical marker genes [16], mammary gland cells were classified into fetal mammary stem cells (fMaSC), basal cells, a mixed mammary precursor/progenitor (MMPr), mature luminal cells (HR^+^ Lum), and alveolar luminal cells (HR^−^ Lum) (Supplementary Figure S5). Analysis of candidate gene expression in each cell subtype revealed that the expression level of *Prox2* was very low, with a small amount of expression in alveolar luminal cells. *Abcd4* was mainly expressed in mature luminal cells and alveolar luminal cells. *Dlst* was mainly expressed in mammary stem cells, mammary precursor/progenitor and alveolar luminal cells, and was also sporadically expressed in mature luminal cells and basal cells (Fig. 1A). *Vrtn* expression was not detected in the sequencing results of any cells (data not shown). Additionally, the expression of *Abcd4*, *Prox2* and *Dlst* was not significantly different among the four periods examined (Fig. 1B).

To determine the dynamic expression pattern of candidate genes during mammary gland development, we collected mammary tissues from 11 critical periods of mammary gland development (Fig. 1C). The expression of the candidate genes at different stages of mammary gland development showed that the expression patterns of *Abcd4* and *Prox2* were relatively similar, with the expression levels gradually increasing with individual development from the embryonic stage, reaching the highest level at late pregnancy (16.5 days of pregnancy), and then decreasing. The expression of the *Dlst* gene was highest at late embryonic stage and puberty. Overall, we characterized the expression patterns of these candidate genes during mammary gland development.

### Effects of genes on proliferation and apoptosis of HC11 cells

**Fig. 2:**
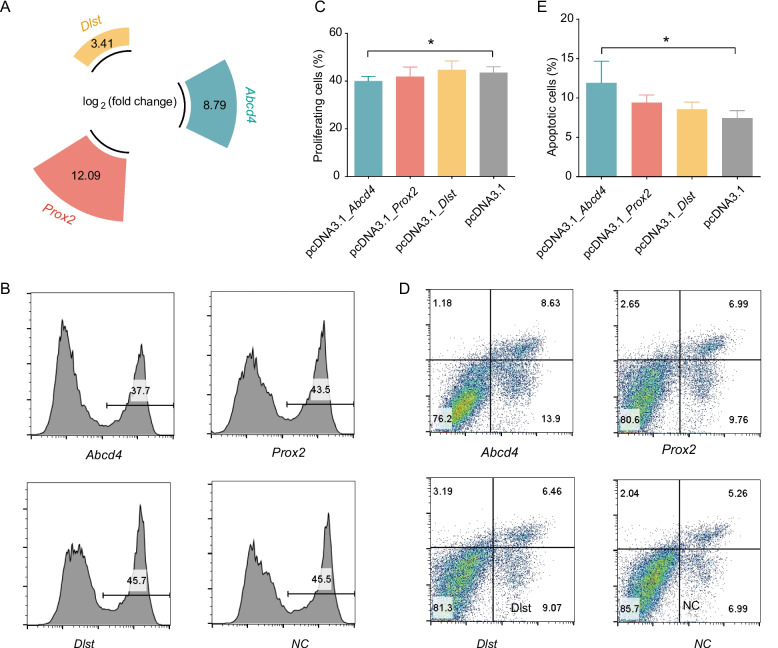
Overexpression of *ABCD4* affects the proliferation and apoptosis of HC11. **A** Fold change analysis of candidate gene overexpression. **B** Flow cytometric EdU analysis of HC11 cells proliferation following overexpression of candidate genes. Only one treatment is shown for each group, and the full data are shown in Supplementary Figure S6. The line segment indicates the area of proliferating cells. **C** Percentage of proliferating cells after candidate gene overexpression treatment. **D** Flow cytometric Annexin-FITC/PI analysis of HC11 cells apoptosis following overexpression of candidate genes. Only one treatment is shown for each group, and the full data are shown in Supplementary Figure S6. **E** Percentage of apoptosis cells after candidate gene overexpression treatment. The values are the means ± SEMs. * represents *P* < 0.05, ** represents *P* < 0.01 and *** represents *P* < 0.001

To assessed whether the candidate genes could affect mammary gland development, we used an overexpression vector to regulate the expression of these genes in HC11 cells. The fold change of average overexpression in *Abcd4*, *Prox2*, and *Dls*t is 444, 4,375, and 11, respectively. The expression of all three genes increased significantly, and the lower the background expression of the candidate genes in the mammary gland, the more significant the overexpression effect (Fig. 2A). Moreover, flow cytometric EdU analysis of HC11 cell proliferation was performed following overexpression of candidate genes. Edu-labeled cells had a higher proportion of green fluorescence (488 fluorescence) positive cells, showing two peaks of green fluorescence negative (weak staining) and positive (strong staining), corresponding to non-proliferating and proliferating cells, respectively. The results suggested that overexpression of *Abcd4* significantly inhibited cell proliferation (Fig. 2B-C). Further observation by flow cytometric Annexin-FITC/PI analysis indicated that overexpression of *Abcd4* significantly promoted HC11 apoptosis (Fig. 2D-E), while overexpression of *Prox2* and *Dlst* had no significant effect on cell proliferation and apoptosis (Fig. 2B-E, Supplementary Figure S6).

### Analysis of the expression profile of HC11 cells overexpressing *Abcd4*

**Fig. 3:**
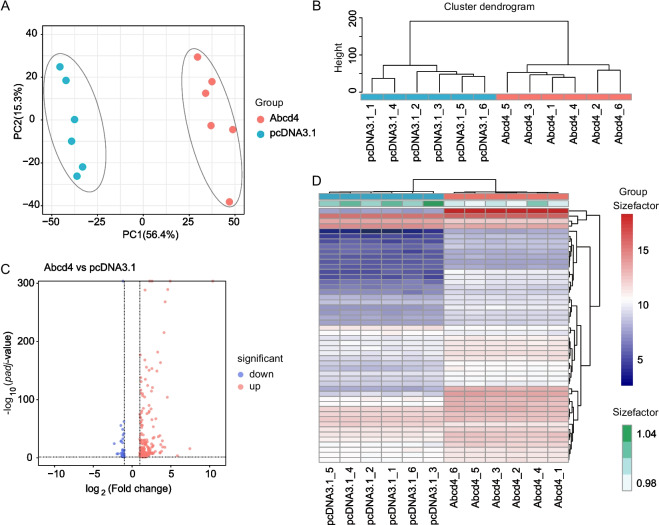
RNA-seq identification of differentially expressed genes following *Abcd4* overexpression. **A** PCA for RNA-seq. The points represent biological replicates. **B** Clustering tree for control and *Abcd4* overexpression samples. **C** Volcano plot of DEGs between control and *Abcd4* overexpression groups. Blue denotes downregulated genes, and red denotes upregulated genes. **D** Heatmap of the top 50 DEGs via hierarchical cluster analysis. Different rows correspond to different genes, and red and blue stripes represent up- and downregulation, respectively

To understand how *Abcd4* affects the proliferation and apoptosis of HC11, RNA-seq was performed to identify differentially expressed genes (DEGs) after overexpression of *Abcd4* in HC11 cells. Principal component analysis (PCA) and cluster dendrogram showed a clear separation between *Abcd4* overexpression and control groups (Fig. 3A, B), suggesting high reproducibility of the transcriptomic profile of *Abcd4* overexpression. A total of 16,633 genes were evaluated (Supplementary Table S2), and compared with the control group, 248 genes showed significant changes in expression in response to the overexpression of *Abcd4*, of which 203 were up-regulated and 45 were down-regulated (Fig. 3C, D).

**Fig. 4:**
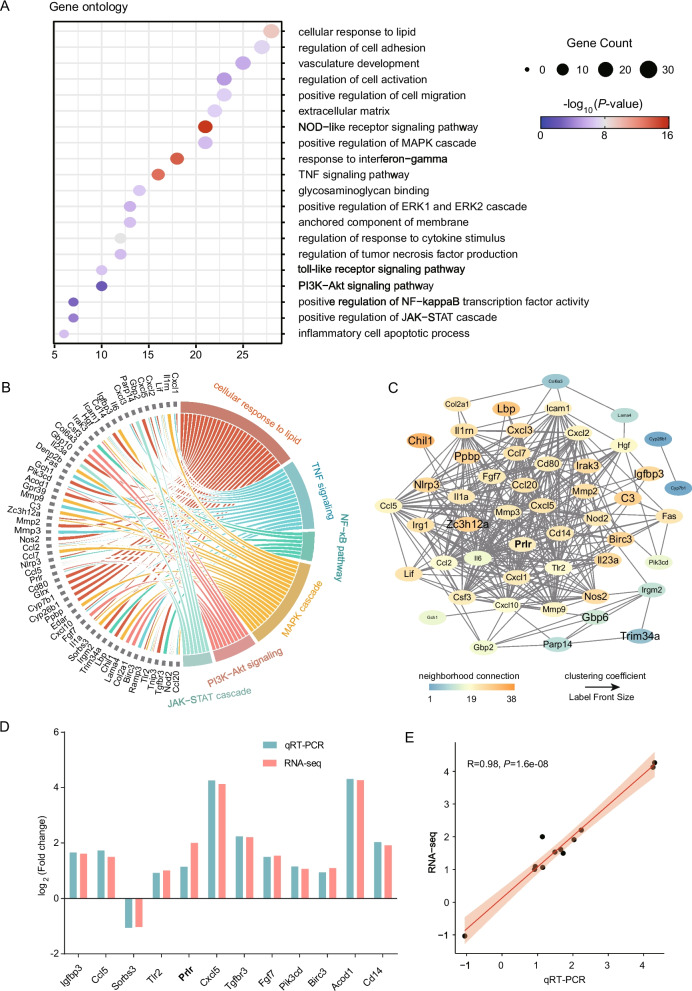
The crucial DEGs associated with mammary gland development. **A** Enrichment analysis of DEGs. **B** Schematic diagram of shared DEGs for important pathways. **C** PPI network of DEGs in important pathways. **D** Comparison of log_2_ (Fold change) of DEGs between qRT-PCR and RNA-seq. **E** Regression analysis of DEGs between qRT-PCR and RNA-seq

To better categorize and characterize the consequences of the *Abcd4* overexpression, enrichment analysis was performed to identify the biological processes by GO terms and KEGG. A total of 20 terms were established by DEGs cluster, among them, the cellular response to lipid (GO:0071396) was enriched by the highest number of DEGs (28 genes) (Fig. 4A). In addition, positive regulation of MAPK cascade (GO:0043410), positive regulation of JAK-STAT cascade (GO:0046427), positive regulation of NF-kappaB transcription factor activity (GO:0051092), PI3K-Akt signaling pathway (ko04151) and TNF signaling pathway (ko04668), which play important roles in mouse mammary gland development, were also significantly enriched (Fig. 4A, Supplementary Table S3). Furthermore, the lipid response, MAPK, TNF, JAK-STAT, NF-κB and PI3K-AKT pathways share a large number of DEGs, i.e., the same gene exists in multiple pathways (Fig. 4B). The protein–protein interaction (PPI) analysis of candidate genes in the above pathways revealed extensive interactions among most of the genes, except for a few genes (Fig. 4C).

Then, we focused on the DEGs within the above pathways, and detected the expression of 12 functionally relevant genes by qRT-PCR, including Igfbp3, Ccl5, Sorbs3, Tlr2, Prlr, Cxcl5, Tgfbr3, Fgf7, Pik3cd, Birc3, Acod1, and Cd14. The gene expression pattern observed in qRT-PCR was consistent with the results of RNA-seq (*R* = 0.98, Fig. 4D, E). Among these genes, the expression of *Prlr* also changed significantly, a crucial regulatory genes in mammary gland development, milk secretion, and functional teat number [21, 22]. These results indicated that *Abcd4* may play a pivotal role during mammary gland development in mice by interacting with *Prlr*.

## Discussion

Altough *ABCD4*, *VRTN*, *PROX2*, and *DLST* were showed to be important candidate genes related to pig teat number [8, 23–25], their functions in mammary gland development are unclear. In this study, we conducted expression and functional analyses on these candidate genes, and discovered that *ABCD4* plays a crucial role in mammary gland development during pregnancy.

*ABCD4* belongs to the ATP-binding cassette transporter superfamily, and is involved in the metabolism of vitamin B12, which is necessary for the formation of red blood cells [26, 27]. Previous studies have not reported its involvement in gland development. However, in our study, we observed that *Abcd4* was mainly expressed in mature luminal cells and alveolar luminal cells during late pregnancy. Alveoli exhibit increased secretory activity during late pregnancy, transforming into lobules that secrete milk during lactation. To prepare for lactation, the mammary gland undergoes gland maturation and alveologenesis, characterized by a substantial increase in ductal branching, proliferating epithelial cells generating alveolar buds, and differentiation into different alveoli [13]. Our findings demonstrated that *Abcd4* significantly inhibits the proliferation of mammary epithelial cells and promote apoptosis. Consequently, we hypothesized that *Abcd4* may influence alveolar luminal cell maturation and lactation.

Prolactin is the main source of lactation capacity during pregnancy, and its biological effects are mediated by its interaction with the prolactin receptor (*Prlr*) [28, 29]. *Prlr* acts both indirectly through the regulation of ovarian progesterone secretion and directly through its effects on mammary epithelial cells, controlling mammary gland development, milk secretion, and the expression of milk protein genes [21, 30, 31]. In this study, *Prlr* expression significantly increased with *Abcd4* overexpression. *Prlr* belongs to the same family as the growth hormone receptor (*Ghr*) and is part of the cytokine receptor superfamily, leading to the activation of many signaling pathways, including Jak/stat, map kinase, and phosphatidylinositol (PI)3 kinase [32]. For example, the Jak2/stat5 cascade has been proven essential for alveolar development during gestation, and activated *Stat5* can crosstalk with PI3K/AKT to jointly mediate the proliferation of alveolar progenitor cells and the survival of their functionally differentiated progeny in the mammary gland [33–36]. Consistent with this, RNA-seq of *Abcd4* overexpressing cells in this study revealed that the differentially expressed genes were significantly enriched in MAPK, JAK-STAT, and PI3K-AKT pathways, and there is a wide range of interactions among these DEGs. These results suggest that *Abcd4* may play a role in mammary gland development and lactation during pregnancy by affecting *Prlr* expression.

From an animal breeding perspective, our prior study identified the most significant locus associated with teat number in the *ABCD4* region [8], a finding supported by recent research [25]. Meanwhile, polymorphisms of the *PRLR* gene have been associated with reproductive traits and milk production traits in pigs, goats, sheep and dairy cattle [37–40]. Specifically, animals with different *PRLR* genotypes show significant differences in the number of functional teats, age at first estrus, litter size, and litter average [22, 41]. Data on litter size and live piglets were not collected in this study, but in swine breeding, a greater number of functional teats usually means a greater number of live piglets [42]. This is because piglets find a specific teat (or pair of teats) within the first few hours of birth and then consistently return to that teat/pair every time they suck, exhibiting "teat fidelity" [43]. Therefore, when the number of litters exceeds the number of teats, teat competition occurs, leading to increased mortality in piglets that cannot have access to normal teats [44, 45]. In this context, we speculate that *ABCD4* plays a role in mammary gland development and lactation during pregnancy, and affects reproductive performance by affecting the number of functional teats in pigs.

In summary, we delved into the expression patterns and regulatory pathways governing four candidate genes implicated in mammary gland development. Notably, our results highlight the potential involvement of *ABCD4* in orchestrating the developmental processes within the mammary gland. By elucidating the roles of these genes, our study contributes to bridging existing knowledge gaps and enhancing comprehension regarding their functions in mammary development. These insights hold promise for the validation of gene functions in other mammalian models and offer valuable guidance for the identification of causative genes and mutations associated with teat number variation in pigs. Nevertheless, it is imperative to acknowledge the limitations of our study. Specifically, the translational relevance of our findings between murine and porcine systems remains ambiguous. Furthermore, the establishment of a functional nexus between the identified candidate genes and teat number in pigs warrants further investigation.

## Conclusions

In this study, we investigated the roles of *VRTN*, *ABCD4*, *PROX2*, and *DLST* in mammary gland development. Notably, we provided novel evidence indicating the pivotal involvement of the *ABCD4* gene in mammary gland development and lactation during pregnancy for the first time. Our findings reveal that *ABCD4* exerts significant effects by inhibiting the proliferation of mammary epithelial cells and facilitating apoptosis through its interaction with *PRLR*. These results underscore the importance of *ABCD4* in mammary gland development.

### Supplementary Information

Additional file 1: Fig. S1. Evolutionary constraint of the *ABCD4* gene. The *ABCD4* gene is conserved in mammals. The data were retrieved from the Ensemble browser (http://asia.ensembl.org/index.html). Fig. S2. Evolutionary constraint of the *VRTN* gene. The *VRTN* gene is conserved in mammals. The data were retrieved from the Ensemble browser (http://asia.ensembl.org/index.html). Fig. S3. Evolutionary constraint of the *PROX2* gene. The *PROX2* gene is conserved in mammals. The data were retrieved from the Ensemble browser (http://asia.ensembl.org/index.html). Fig. S4. Evolutionary constraint of the *DLST* gene. The *DLST* gene is conserved in mammals. The data were retrieved from the Ensemble browser (http://asia.ensembl.org/index.html). Fig. S5. Expression of marker genes related to mammary cell subtypes. Fig. S6. Flow cytometric analysis of HC11 cells proliferation and apoptosis following overexpression of candidate genes.

Additional file 2: Table S1. The primer sequences used in this study. Table S2. Gene expression profile of HC11 cells overexpressing Abcd4. Table S3. Enrichment analysis of DEGs.

Additional file 3. The codes for scRNA-seq and RNA-seq analysis in this study.

## Data Availability

All of the sequencing data in this study have been deposited into NCBI and can be accessed via accession PRJNA999479.

## References

[1] Fu NY, Nolan E, Lindeman GJ, Visvader JE (2020). Stem Cells and the Differentiation Hierarchy in Mammary Gland Development. Physiol Rev.

[2] Bach K, Pensa S, Grzelak M, Hadfield J, Adams DJ, Marioni JC, Khaled WT (2017). Differentiation dynamics of mammary epithelial cells revealed by single-cell RNA sequencing. Nat Commun.

[3] Veltmaat JM (2017). Prenatal Mammary Gland Development in the Mouse: Research Models and Techniques for Its Study from Past to Present. Methods Mol Biol.

[4] Richert MM, Schwertfeger KL, Ryder JW, Anderson SM (2000). An atlas of mouse mammary gland development. J Mammary Gland Biol Neoplasia.

[5] Robinson GW (2007). Cooperation of signalling pathways in embryonic mammary gland development. Nat Rev Genet.

[6] Gray GK, Girnius N, Kuiken HJ, Henstridge AZ, Brugge JS (2023). Single-cell and spatial analyses reveal a tradeoff between murine mammary proliferation and lineage programs associated with endocrine cues. Cell Rep.

[7] Paine IS, Lewis MT (2017). The Terminal End Bud: the Little Engine that Could. J Mammary Gland Biol Neoplasia.

[8] Yang R, Guo X, Zhu D, Tan C, Bian C, Ren J, Huang Z, Zhao Y, Cai G, Liu D (2021). Accelerated deciphering of the genetic architecture of agricultural economic traits in pigs using a low-coverage whole-genome sequencing strategy. Gigascience..

[9] Zhuang Z, Ding R, Peng L, Wu J, Ye Y, Zhou S, Wang X, Quan J, Zheng E, Cai G (2020). Genome-wide association analyses identify known and novel loci for teat number in Duroc pigs using single-locus and multi-locus models. BMC Genomics.

[10] van Son M, Lopes MS, Martell HJ, Derks MFL, Gangsei LE, Kongsro J, Wass MN, Grindflek EH, Harlizius B (2019). A QTL for Number of Teats Shows Breed Specific Effects on Number of Vertebrae in Pigs: Bridging the Gap Between Molecular and Quantitative Genetics. Front Genet.

[11] Moscatelli G, Dall'Olio S, Bovo S, Schiavo G, Kazemi H, Ribani A, Zambonelli P, Tinarelli S, Gallo M, Bertolini F (2020). Genome-wide association studies for the number of teats and teat asymmetry patterns in Large White pigs. Anim Genet.

[12] Hurley WL (2019). Review: Mammary gland development in swine: embryo to early lactation. Animal.

[13] Macias H, Hinck L (2012). Mammary gland development. Wiley Interdiscip Rev Dev Biol.

[14] Wang W, Wang S, Wang H, Zheng E, Wu Z, Li Z (2024). Protein Dynamic Landscape during Mouse Mammary Gland Development from Virgin to Pregnant, Lactating, and Involuting Stages. J Agric Food Chem.

[15] Inman JL, Robertson C, Mott JD, Bissell MJ (2015). Mammary gland development: cell fate specification, stem cells and the microenvironment. Development.

[16] Giraddi RR, Chung CY, Heinz RE, Balcioglu O, Novotny M, Trejo CL, Dravis C, Hagos BM, Mehrabad EM, Rodewald LW (2018). Single-Cell Transcriptomes Distinguish Stem Cell State Changes and Lineage Specification Programs in Early Mammary Gland Development. Cell Rep.

[17] Stuart T, Butler A, Hoffman P, Hafemeister C, Papalexi E, Mauck WM, Hao Y, Stoeckius M, Smibert P, Satija R (2019). Comprehensive integration of single-cell data. Cell.

[18] Bolger AM, Lohse M, Usadel B (2014). Trimmomatic: a flexible trimmer for Illumina sequence data. Bioinformatics.

[19] Kim D, Langmead B, Salzberg SL (2015). HISAT: a fast spliced aligner with low memory requirements. Nat Methods.

[20] Love MI, Huber W, Anders S (2014). Moderated estimation of fold change and dispersion for RNA-seq data with DESeq2. Genome Biol.

[21] Kelly PA, Bachelot A, Kedzia C, Hennighausen L, Ormandy CJ, Kopchick JJ, Binart N (2002). The role of prolactin and growth hormone in mammary gland development. Mol Cell Endocrinol.

[22] van Rens BT, van der Lende T (2002). Litter size and piglet traits of gilts with different prolactin receptor genotypes. Theriogenology.

[23] Tan C, Wu Z, Ren J, Huang Z, Liu D, He X, Prakapenka D, Zhang R, Li N, Da Y (2017). Genome-wide association study and accuracy of genomic prediction for teat number in Duroc pigs using genotyping-by-sequencing. Genet Sel Evol.

[24] Ren DR, Ren J, Ruan GF, Guo YM, Wu LH, Yang GC, Zhou LH, Li L, Zhang ZY, Huang LS (2012). Mapping and fine mapping of quantitative trait loci for the number of vertebrae in a White Duroc x Chinese Erhualian intercross resource population. Anim Genet.

[25] Yang L, Li X, Zhuang Z, Zhou S, Wu J, Xu C, Ruan D, Qiu Y, Zhao H, Zheng E (2023). Genome-Wide Association Study Identifies the Crucial Candidate Genes for Teat Number in Crossbred Commercial Pigs. Animals (Basel)..

[26] Choi YM, Kim YI, Choi JH, Bhandari S, Nam IK, Hong K, Kwak S, So HS, Park DS, Kim CH (2019). Loss of abcd4 in zebrafish leads to vitamin B(12)-deficiency anemia. Biochem Biophys Res Commun.

[27] Coelho D, Kim JC, Miousse IR, Fung S, du Moulin M, Buers I, Suormala T, Burda P, Frapolli M, Stucki M (2012). Mutations in ABCD4 cause a new inborn error of vitamin B12 metabolism. Nat Genet.

[28] Bole-Feysot C, Goffin V, Edery M, Binart N, Kelly PA (1998). Prolactin (PRL) and its receptor: actions, signal transduction pathways and phenotypes observed in PRL receptor knockout mice. Endocr Rev.

[29] Feng P, Wu J, Ren Y, Zhang L, Cao J, Yang L (2022). Early pregnancy regulates the expression of prolactin and its receptor in the thymus, the liver, the spleen and lymph nodes in sheep. Domest Anim Endocrinol.

[30] Freeman ME, Kanyicska B, Lerant A, Nagy G (2000). Prolactin: structure, function, and regulation of secretion. Physiol Rev.

[31] Viitala S, Szyda J, Blott S, Schulman N, Lidauer M, Maki-Tanila A, Georges M, Vilkki J (2006). The role of the bovine growth hormone receptor and prolactin receptor genes in milk, fat and protein production in Finnish Ayrshire dairy cattle. Genetics.

[32] Clevenger CV, Freier DO, Kline JB (1998). Prolactin receptor signal transduction in cells of the immune system. J Endocrinol.

[33] Watson CJ, Neoh K (2008). The Stat family of transcription factors have diverse roles in mammary gland development. Semin Cell Dev Biol.

[34] Cui Y, Riedlinger G, Miyoshi K, Tang W, Li C, Deng CX, Robinson GW, Hennighausen L (2004). Inactivation of Stat5 in mouse mammary epithelium during pregnancy reveals distinct functions in cell proliferation, survival, and differentiation. Mol Cell Biol.

[35] Tian M, Qi Y, Zhang X, Wu Z, Chen J, Chen F, Guan W, Zhang S (2020). Regulation of the JAK2-STAT5 Pathway by Signaling Molecules in the Mammary Gland. Frontiers in cell and developmental biology.

[36] Rädler PD, Wehde BL, Wagner KU (2017). Crosstalk between STAT5 activation and PI3K/AKT functions in normal and transformed mammary epithelial cells. Mol Cell Endocrinol.

[37] Terman A (2005). Effect of the polymorphism of prolactin receptor (PRLR) and leptin (LEP) genes on litter size in Polish pigs. J Anim Breed Genet.

[38] Chu MX, Mu YL, Fang L, Ye SC, Sun SH (2007). Prolactin receptor as a candidate gene for prolificacy of small tail han sheep. Anim Biotechnol.

[39] Hou JX, An XP, Song YX, Wang JG, Ma T, Han P, Fang F, Cao BY (2013). Combined effects of four SNPs within goat PRLR gene on milk production traits. Gene.

[40] El-Magd MA, Fathy A, Kahilo KA, Saleh AA, El Sheikh AI, Al-Shami S, El-Komy SM (2021). Polymorphisms of the PRLR Gene and Their Association with Milk Production Traits in Egyptian Buffaloes. Animals (Basel)..

[41] El-Shorbagy HM, Abdel-Aal ES, Mohamed SA, El-Ghor AA (2022). Association of PRLR, IGF1, and LEP genes polymorphism with milk production and litter size in Egyptian Zaraibi goat. Trop Anim Health Prod.

[42] Kim JS, Jin DI, Lee JH, Son DS, Lee SH, Yi YJ, Park CS (2005). Effects of teat number on litter size in gilts. Anim Reprod Sci.

[43] Skok J (2018). On the presence and absence of suckling order in polytocous mammals. Behav Processes.

[44] Kobek-Kjeldager C, Moustsen VA, Theil PK, Pedersen LJ (2020). Effect of litter size, milk replacer and housing on production results of hyper-prolific sows. Animal.

[45] Rutherford KMD, Baxter EM, D’Eath RB, Turner SP, Arnott G, Roehe R, Ask B, SandØe P, Moustsen VA, Thorup F (2023). The welfare implications of large litter size in the domestic pig I: biological factors. Anim Welf.

