# Supplementary material for: ABCD4 is associated with mammary gland development in mammals
Source: BMC Genomics. 2024 May 20;25:494. doi: 10.1186/s12864-024-10398-9 (PMC11103957; doi:10.1186/s12864-024-10398-9)
Supplement: Supplementary file 1 — Additional file 1: Fig. S1. Evolutionary constraint of the ABCD4 gene. The ABCD4 gene is conserved in mammals. The data were retrieved from the Ensemble browser (http://asia.ensembl.org/index.html). Fig. S2. Evolutionary constraint of the VRTN gene. The VRTN gene is conserved in mammals. The data were retrieved from the Ensemble browser (http://asia.ensembl.org/index.html). Fig. S3. Evolutionary constraint of the PROX2 gene. The PROX2 gene is conserved in mammals. The data were retrieved from the Ensemble browser (http://asia.ensembl.org/index.html). Fig. S4. Evolutionary constraint of the DLST gene. The DLST gene is conserved in mammals. The data were retrieved from the Ensemble browser (http://asia.ensembl.org/index.html). Fig. S5. Expression of marker genes related to mammary cell subtypes. Fig. S6. Flow cytometric analysis of HC11 cells proliferation and apoptosis following overexpression of candidate genes. [file 12864_2024_10398_MOESM1_ESM.docx]

***ABCD4* is associated with mammary gland development in mammals**

Xiaoli Guo, Chengcheng Zhao, Ruifei Yang, Yuzhe Wang, and Xiaoxiang Hu

*BMC Genomics* journal


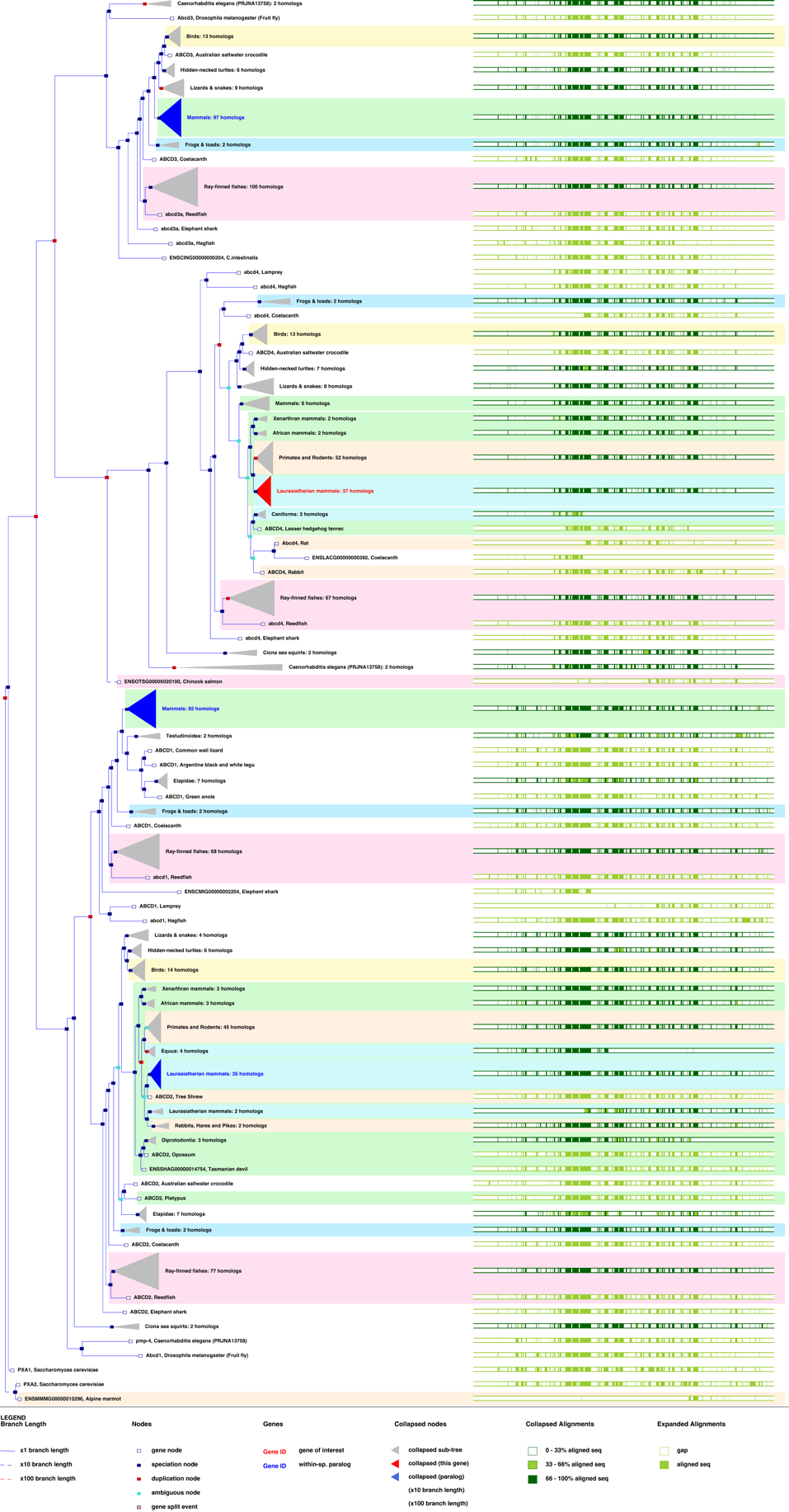


**Supplementary Figure S1** Evolutionary constraint of the *ABCD4* gene. The *ABCD4* gene is conserved in mammals. The data were retrieved from the Ensemble browser (<http://asia.ensembl.org/index.html>).


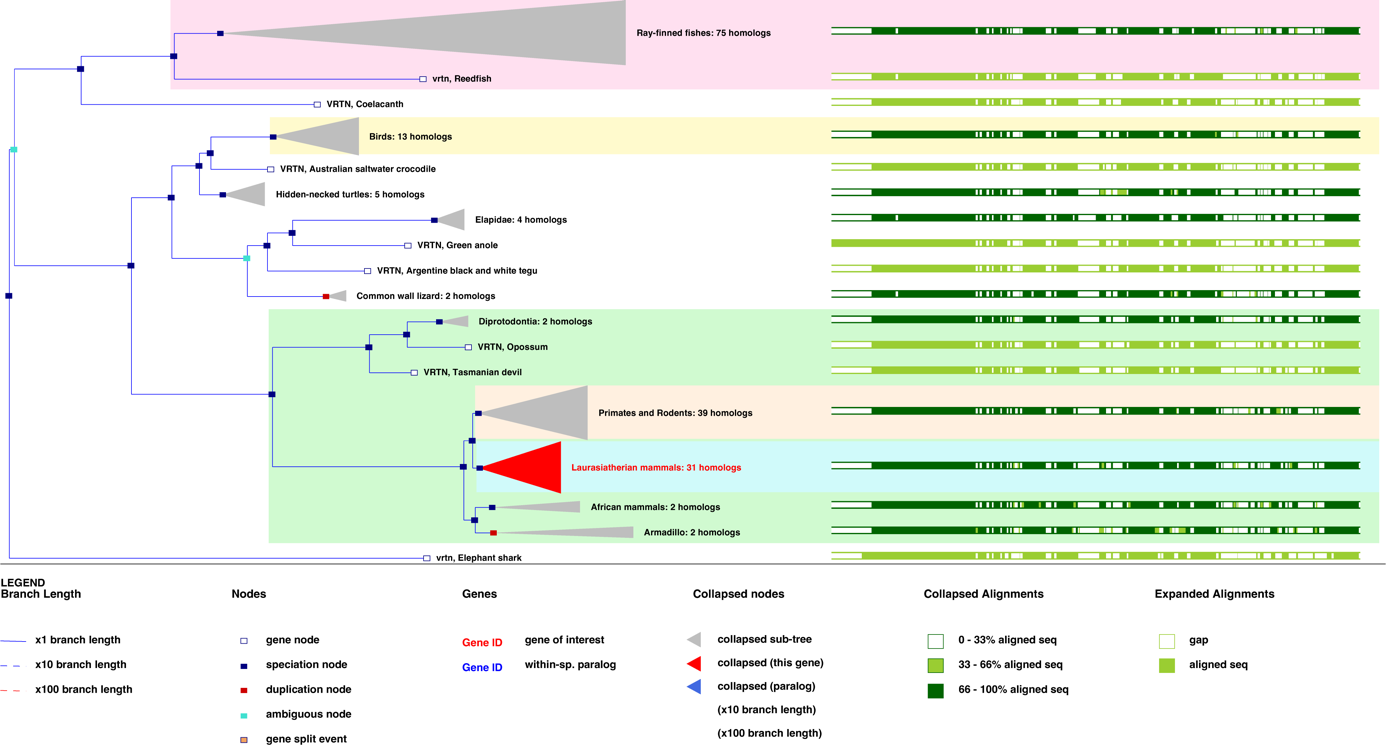


**Supplementary Figure S2** Evolutionary constraint of the *VRTN* gene. The *VRTN* gene is conserved in mammals. The data were retrieved from the Ensemble browser (<http://asia.ensembl.org/index.html>).


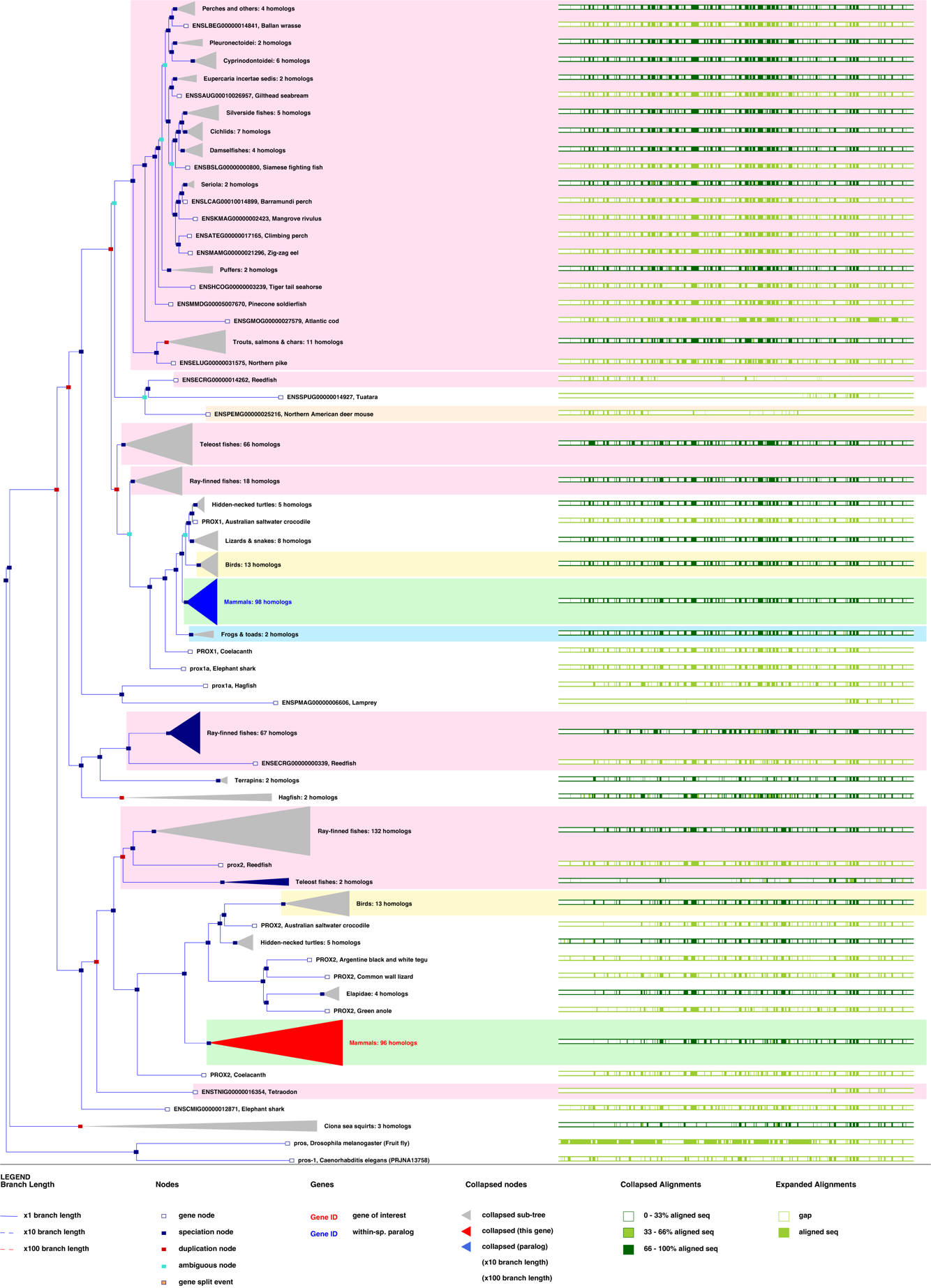


**Supplementary Figure S3** Evolutionary constraint of the *PROX2* gene. The *PROX2* gene is conserved in mammals. The data were retrieved from the Ensemble browser (<http://asia.ensembl.org/index.html>).


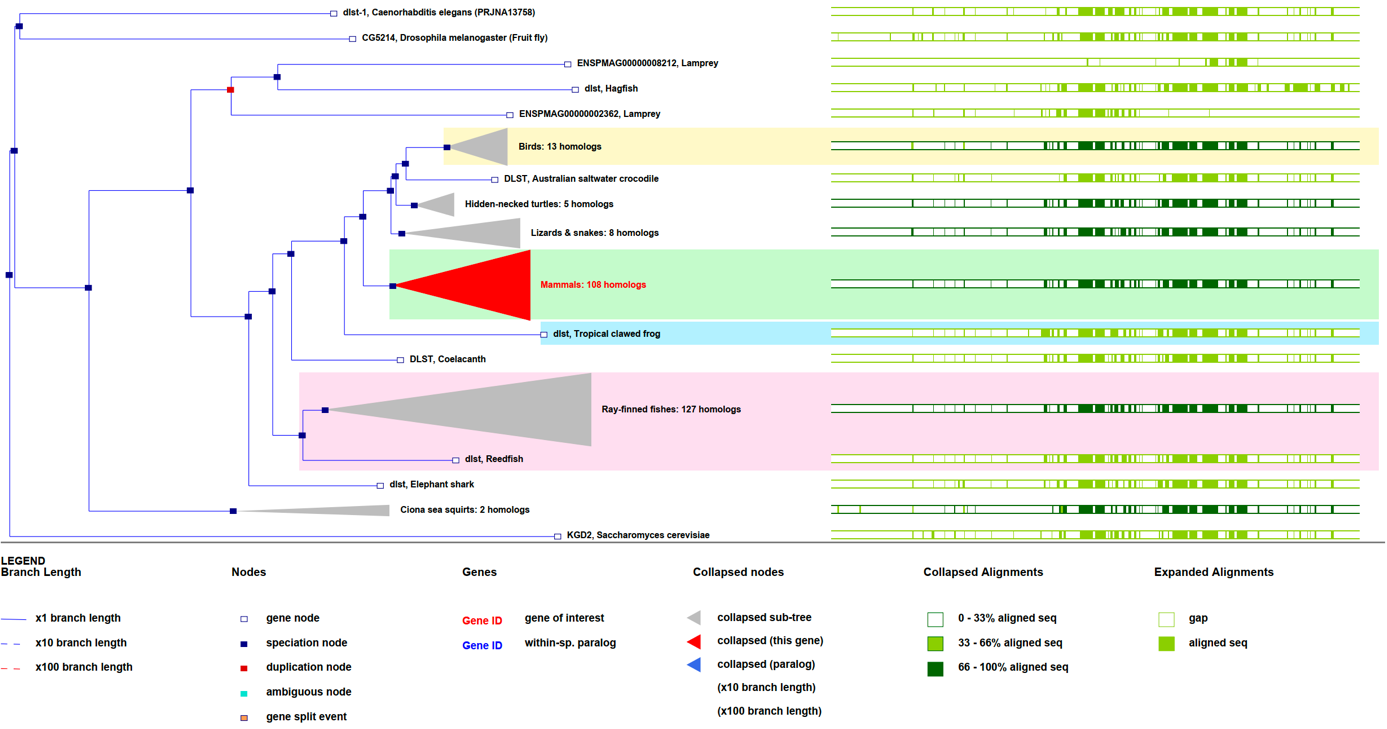


**Supplementary Figure S4** Evolutionary constraint of the *DLST* gene. The *DLST* gene is conserved in mammals. The data were retrieved from the Ensemble browser (<http://asia.ensembl.org/index.html>).


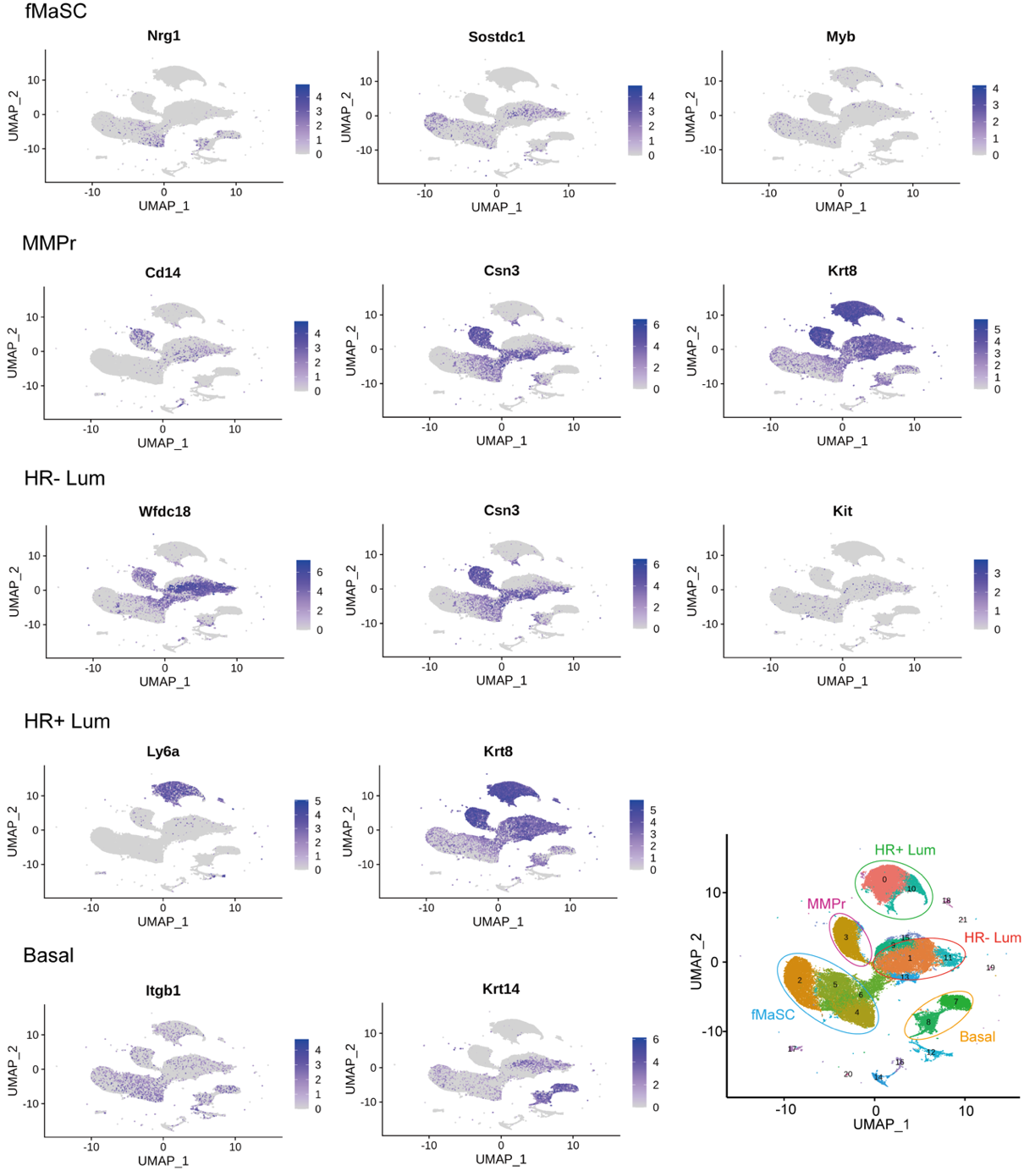


**Supplementary Figure S5** Expression of marker genes related to mammary cell subtypes


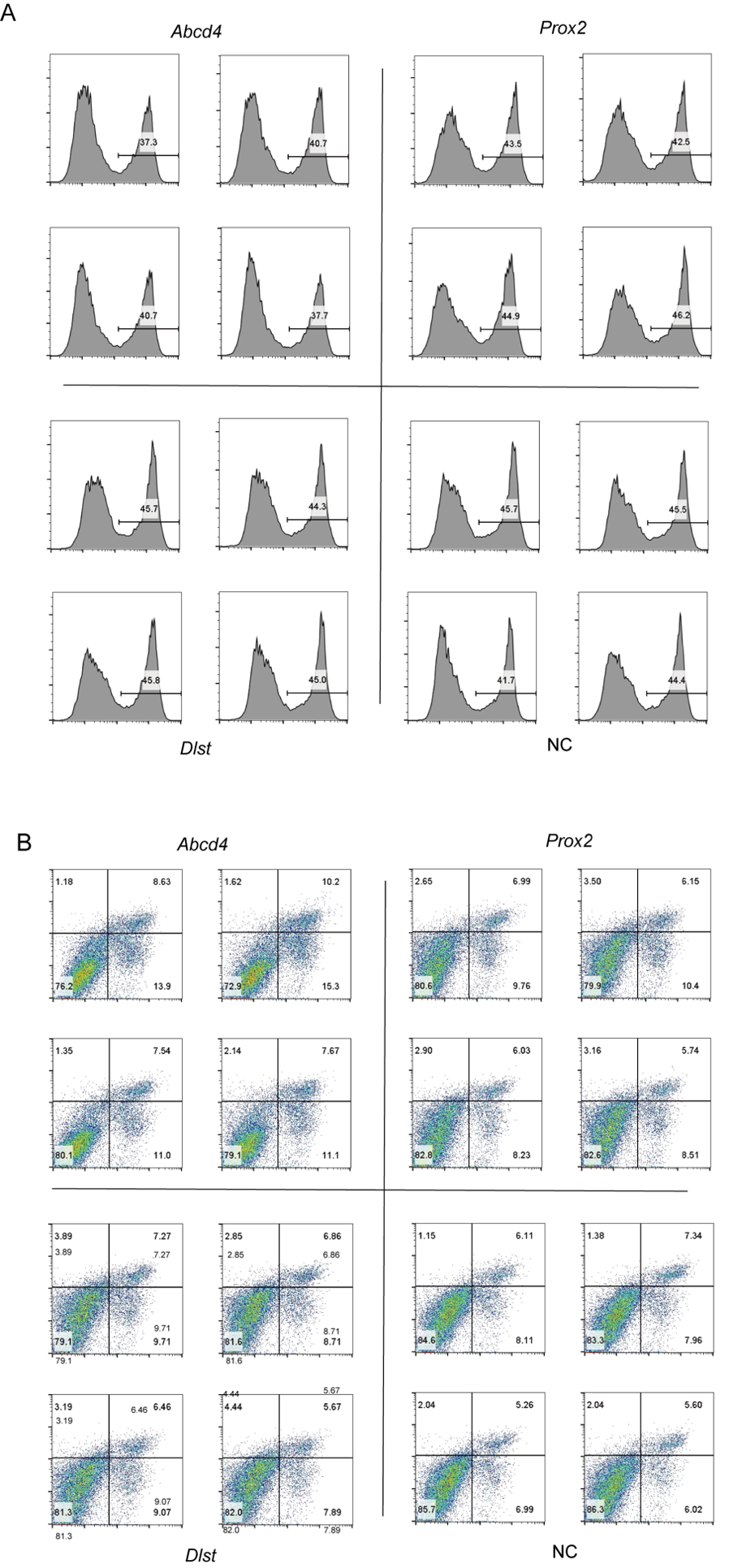


**Supplementary Figure S6** Flow cytometric analysis of HC11 cells proliferation and apoptosis following overexpression of candidate genes
